# Peeling Back the Layers: Sloughing Esophagitis in a Teenager with Chronic Vomiting

**DOI:** 10.1097/PG9.0000000000000376

**Published:** 2023-11-13

**Authors:** Jessica V. Baran, Rowan O’Flanagan, Carolena Trocchia, Jerry M. Brown, Johnny Nguyen, Sara Karjoo, Michael J. Wilsey

**Affiliations:** From the * Office of Medical Education, Florida Atlantic University Charles E. Schmidt College of Medicine, Boca Raton, FL; † Department of Biophysics, Johns Hopkins University, Baltimore, MD; ‡ Office of Medical Education, Johns Hopkins All Children’s Hospital, St. Petersburg, FL; § Department of Pathology, Johns Hopkins All Children’s Hospital, St. Petersburg, FL; ∥ Department of Pediatric Gastroenteroleogy, Johns Hopkins All Children’s Hospital, St. Petersburg, FL.

**Keywords:** esophagitis dissecans superficialis, endoscopy, pediatric gastroenterology, esophageal disorder

## Abstract

Esophagitis dissecans superficialis (EsoDS) is a rare condition characterized by the shedding of superficial esophageal epithelium. Limited data exists on EsoDS in the pediatric population. We present a case of a 17-year-old female with chronic nausea and vomiting diagnosed with EsoDS. Endoscopy revealed esophageal mucosal sloughing, and histology confirmed esophagitis with mucosal necrosis. EsoDS is underrecognized, and its association with psychoactive medications remains unclear. Fortunately, EsoDS cases tend to resolve spontaneously without complications. Awareness of EsoDS is essential, and further research is needed to understand its prevalence and outcomes in pediatric patients.

## INTRODUCTION

Esophagitis dissecans superficialis (EsoDS), or sloughing esophagitis, is a desquamative disorder characterized by the shedding of superficial esophageal epithelium, presenting with variable clinical features. Endoscopically, it manifests as superficial peeling of epithelial sheets while histologically displaying a 2-toned appearance with coagulative necrosis affecting the superficial layers (1–4). Clinically, patients can present as asymptotic or with nonspecific symptoms, including dysphagia, nausea, vomiting, odynophagia hematemesis, and epigastric pain with gradual progression (1). Limited data exists regarding this condition in the pediatric population (1,2). We report a 17-year-old female presenting with chronic symptoms of nausea and vomiting diagnosed with EsoDS.

## CASE REPORT

A 17-year-old female with a history of anxiety and depression presented with progressive symptoms of nausea, vomiting, and constipation that have been ongoing for approximately 2–3 years. Nausea occurs daily without identifiable triggers or alleviating factors. Vomiting episodes, occurring 1–2 times per week, occasionally provide temporary relief. No specific food correlations were identified through a food diary, and she maintains a regular diet. The patient experiences frequent headaches, potentially less frequent than nausea. Abdominal pain is absent, while burping is noted. The patient reports a decreased appetite, consuming 1–2 meals daily, and no significant weight loss. Her current medications include fluoxetine, guanfacine, and an oral contraceptive.

**FIGURE 1.**
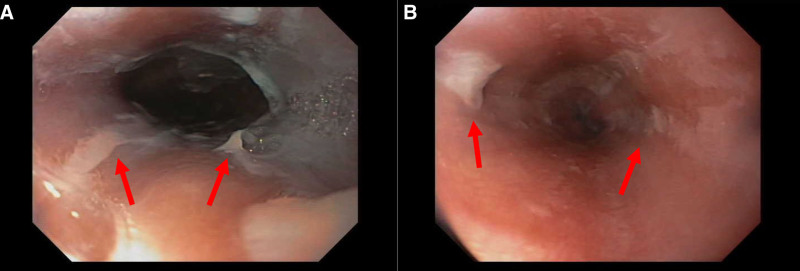
Endoscopy image showing sloughing esophagitis (red arrows) in which there are vertical lines or patches of detaching white superficial squamous mucosa in the mid-esophagus (A) and distal esophagus (B).

**FIGURE 2.**
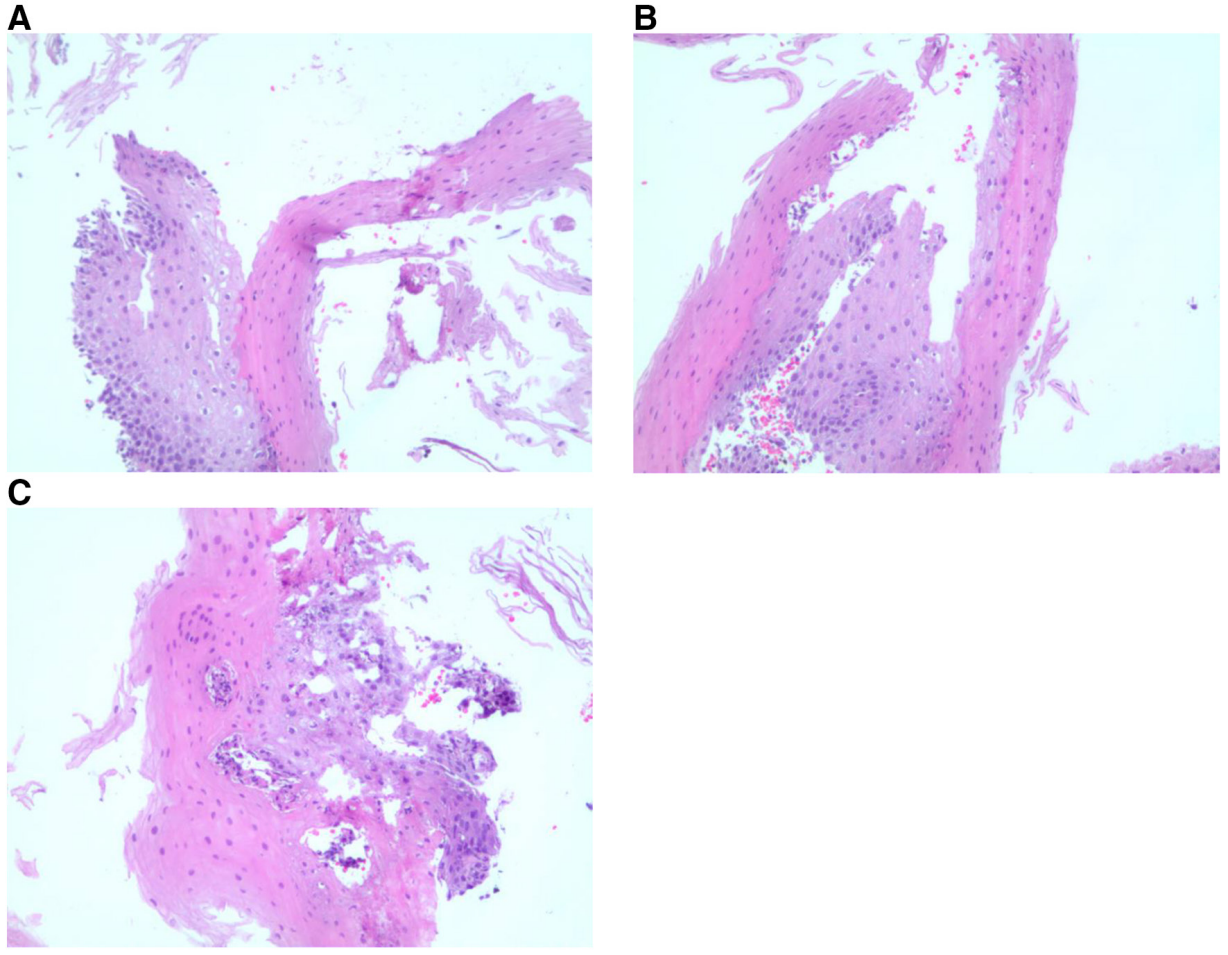
A and B) Medium power (10× magnification). H&E sections of esophagus showed a “two-toned” appearance with superficial separation of the squamous epithelial layer above the basal cell layer, without inflammation or microorganisms. C) Medium power (10× magnification). H&E section showing esophageal mucosa with focal areas of necrosis and degeneration of the squamous mucosa. Note the “two-toned” appearance and the conspicuous lack of inflammatory cells.

Upper endoscopy revealed sloughing of the mucosa with loss of vascular pattern, concerning esophagitis (Fig. 1). No discrete ulcers were noted. Histology revealed chronic esophagitis with partial superficial squamous mucosal necrosis and sloughing (Fig. 2).

## DISCUSSION

EsoDS is an underrecognized esophageal diagnosis (3,4), particularly in pediatric patients (1,2). The use of psychoactive medications has been associated with EsoDS diagnosis (2,3). Although the pathogenesis for this association has not yet been explained, researchers suspect that selective serotonin reuptake inhibitors, bisphosphonates, nonsteroidal anti-inflammatory drugs, and doxycycline can irritate the esophageal lining, thus predisposing patients to EsoDS (5–9). Fortunately, EsoDS cases generally resolve spontaneously without long-term complications (1–4). When endoscopic and histopathologic data support an EsoDS diagnosis, the need for extensive workup and medication interventions may be reduced (1–4).

Given its underrecognized nature, endoscopists should be familiar with EsoDS (1). Chronic irritation or injury to the esophageal lining, including gastroesophageal reflux disease, eosinophilic esophagitis, and food impaction, can be potential precursors to EsoDS development (1,3). Notably, pediatric EsoDS appears to be an incidental finding, lacking significant morbidity or mortality implications (1,2). Although there are some reported cases with follow-up endoscopy, no specific guidelines regarding surveillance endoscopy for patients with this condition have been established in the existing literature (8). Counseling patients should aim to provide comprehensive information about the condition, its underlying causes, and recommended management strategies. Additionally, counseling should also encompass possible risk factors and ways to decrease exposure to them, such as hot beverages, medications (selective serotonin reuptake inhibitors, doxycycline, bisphosphonate, and nonsteroidal anti-inflammatory drugs), autoimmune bullous dermatosis (eg, pemphigus vulgaris and mucous membrane pemphigoid), esophageal iatrogenic injury (sclerotherapy, band ligation, dilatation, and mediastinal radiation) and heavy smoking (8,9). Further research is warranted to enhance our understanding of EsoDS’s prevalence, clinical features, and outcomes in the pediatric population.

## ACKNOWLEDGMENTS

Informed consent was obtained from the parents to publish this work.
